# Challenges of HIV Lymphoma Clinical Trials in Africa: Lessons From the AIDS Malignancy Consortium 068 Study

**DOI:** 10.1200/GO.20.00152

**Published:** 2020-07-07

**Authors:** Robert M. Strother, Satish Gopal, Meg Wirth, Amy Chadburn, Ariela Noy, Ethel Cesarman, Jeannette Y. Lee, Scot C. Remick, Naftali Busakhala, Bongani Kaimila, Elson Mberi, Ntokozo Ndlovu, Abrahams Omoding, Susan E. Krown

**Affiliations:** ^1^ University of Otago, Christchurch, New Zealand; ^2^ Center for Global Health, National Cancer Institute, Rockville, MD; ^3^ The Emmes Corporation, Callaway, MD; ^4^ Weill Cornell Medical College, New York, NY; ^5^ Memorial Sloan Kettering Cancer Center, New York, NY; ^6^ University of Arkansas for Medical Sciences, Little Rock, AR; ^7^ Maine Medical Center, Portland, ME, and Tufts University, Boston, MA; ^8^ Moi Teaching and Referral Hospital, Eldoret, Kenya; ^9^ University of North Carolina Project-Malawi, Lilongwe, Malawi; ^10^ University of Zimbabwe, Harare, Zimbabwe; ^11^ Uganda Cancer Institute, Kampala, Uganda; ^12^ AIDS Malignancy Consortium, New York, NY

## Abstract

The purpose of this article is to describe lessons from the first lymphoma clinical trial conducted by the AIDS Malignancy Consortium (AMC) in sub-Saharan Africa (SSA). AMC-068 was a randomized phase II comparison of intravenous versus oral chemotherapy for HIV-positive diffuse large B-cell lymphoma. Opening in 2016, AMC-068 planned to enroll 90 patients (45 per arm) in Kenya, Malawi, Uganda, and Zimbabwe over 24 months and follow patients for 24 months to assess overall survival. In 2018, the study closed after screening 42 patients but enrolling only 7. Challenges occurred during protocol development, pre-activation, and postactivation. During protocol development (2011-2012), major obstacles were limited baseline data to inform study design; lack of consensus among investigators and approving bodies regarding appropriateness of the oral regimen and need for randomized comparison with cyclophosphamide, doxorubicin, vincristine, and prednisone; and heterogeneity across sites in local standards for diagnosis, staging, and treatment. During pre-activation (2012-2016), challenges included unexpected length and layers of regulatory approval across multiple countries, need to upgrade pathology capacity at sites, need to augment existing chemotherapy infusion capacity at sites, and procurement issues for drugs and supplies. Finally, during postactivation (2016-2018), challenges included long delays between symptom onset and screening entry for many patients, leading to compromised performance status and organ function; other patient characteristics that frequently led to exclusion, including high tumor proliferative index or other pathologic features that were disallowed; and costs of routine diagnostic procedures often being borne by patients, which also contributed to pre-enrollment delays. Lessons from AMC-068 are being applied to the design and conduct of new AMC lymphoma trials in SSA, and the study has contributed to a strong operational foundation that will support innovative clinical trials in the future.

## BACKGROUND

The coexistence of HIV and cancer is a growing global health issue as more people living with HIV/AIDS (PLWHA) gain access to combined antiretroviral therapy (cART). In the cART era, PLWHA in high-income countries show a 10- to 20-fold higher incidence of AIDS-defining non-Hodgkin lymphomas (NHL), such as diffuse large B-cell lymphoma (DLBCL), than the general population.^1^ In such settings, treatment with cART, rituximab, and combination chemotherapy has resulted in outcomes for AIDS-related (AR) NHL (AR-NHL) comparable to those for HIV-negative individuals.^2^ However, the successful use of such treatments in PLWHA requires supportive measures to minimize treatment-related mortality and maximize overall survival (OS).

Countries in sub-Saharan Africa (SSA) bear the highest burdens of both HIV infection and AR-NHL. However, in most SSA settings, delivering complex chemotherapy regimens with adequate supportive care is difficult because of limited resources.^3^ Scarce resources also impede accurate pathologic classification, often resulting in suboptimal treatment, with many morphologically high-grade lymphomas, including Burkitt lymphoma, being treated with cyclophosphamide, doxorubicin, vincristine, and prednisone (CHOP).^4,5^ A recent prospective registry study in Malawi showed that concurrent cART and CHOP could be delivered safely and with efficacy similar to that in resource-rich environments.^6,7^ However, these data were generated from a single urban referral center with strong external academic partnerships, with uncertain generalizability to other SSA settings. Although numerous efforts are ongoing to strengthen the cancer diagnostic and treatment infrastructure in SSA, patient access to centers with robust capabilities for administering intravenous chemotherapy with adequate supportive care remain limited. These realities have prompted interest in developing effective, less intensive treatments for AR-NHL.

The oral chemotherapy regimen (oCT), developed by Remick et al^8^ in the early 1990s, was used by Mwanda et al^9^ in SSA in the 2000s to address some of these challenges. The regimen, comprising lomustine, etoposide, cyclophosphamide, and procarbazine, was designed to avoid the need for intravenous administration, while minimizing myelosuppression. Of 49 participants recruited between 2001 and 2005 in Kenya and Uganda, 40 (82%) were assessable, with an overall response rate of 78%. Additionally, only 3 treatment-related deaths occurred, and only 5% of chemotherapy cycles required dose reduction for neutropenia.

Although these preliminary results were encouraging, this trial had several important limitations. Only 18 of 49 participants (37%) received concurrent cART, which does not reflect current HIV treatment patterns in SSA. Staging and response assessment were limited in most cases to clinical examination, chest radiograph, and abdominal ultrasound, with 16 participants (31%) classified as stage I or II. Initial diagnosis was based on hematoxylin and eosin (H&E) staining, but subsequent review of a subset of patients showed a range of morphologic subtypes, with only 25% being DLBCL. Given these limitations and interest in comparing the oCT regimen with CHOP, the regional standard-of-care (SOC), the AIDS Malignancy Consortium (AMC) developed a randomized, phase II trial of CHOP versus oCT in AR-DLBCL.

The trial, entitled Randomized, Phase II Trial of CHOP Versus Oral Chemotherapy With Concomitant Antiretroviral Therapy in Patients With HIV-Associated Lymphoma in Sub-Saharan Africa (AMC-068; ClinicalTrials.gov identifier: NCT01775475), was the first SSA therapeutic trial developed by the AMC and was eventually opened to accrual at 4 SSA AMC sites. Although the study was ultimately terminated early because of slow accrual, several important lessons were learned, and this experience has informed the expanding AMC international program. In this article, we review the AMC-068 experience, describing specific challenges faced by the investigative team, solutions identified, and how the lessons learned are being applied to future AMC clinical trials in SSA.

## AMC-068 PROTOCOL DESIGN AND TIMELINE

AMC-068 was developed and approved by the AMC and National Cancer Institute (NCI) in 2012. The target population was PLWHA receiving effective cART who had biopsy-proven, untreated stage III or IV DLBCL. Participants were randomly assigned to receive CHOP or oCT. Relevant inclusion criteria were ability to provide informed consent; age ≥ 18 years; confirmed HIV infection; biopsy-proven DLBCL, defined by large-cell morphology on H&E staining, CD20, or Pax5 positivity based on immunostaining, and a proliferation rate of ≤ 90% determined by immunostaining for Ki67; Eastern Cooperative Oncology Group (ECOG) performance status 0-3; estimated life expectancy > 6 weeks; adequate bone marrow, renal, and liver function; negative CSF cytology within 4 weeks; and cART consistent with national guidelines. The proliferation rate specification was included to avoid potential administration of low-intensity treatment of Burkitt and other high-grade, highly proliferative B-cell lymphoma subtypes. Relevant exclusion criteria were > 10 days of corticosteroid treatment more than physiologic replacement, evidence of CNS lymphoma, and active infections. Participants were stratified by CD4 count (< or ≥ 100) and ECOG performance status (< 2 *v* ≥ 2). The primary outcome was OS. Secondary outcomes included overall response rate, progression-free survival, and safety and tolerance of the respective regimens. Exploratory objectives included treatment completion, adherence, and effects of protocol treatment on HIV control. Details of the drug regimens are provided in Table 1.

**TABLE 1:**
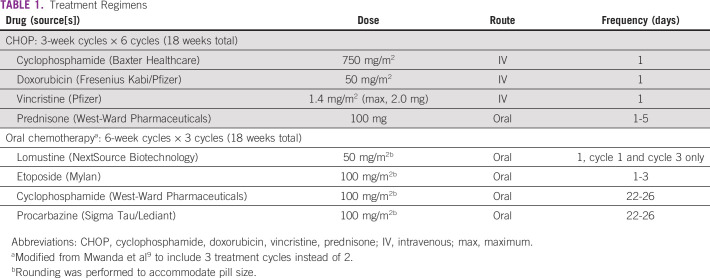
Treatment Regimens

The target sample size of 90 evaluable participants, 45 in each arm, was based on the null hypothesis that CHOP and oCT did not differ with respect to OS, with the alternative hypothesis that the median OS duration would be 18 months for CHOP and 12 months for the oral regimen. Based on estimates of the number of potentially eligible participants at the participating AMC sites, accrual was expected to be completed in 24 months, with an additional 24 months of follow-up.

Although originally designed in 2011-2012, the study did not open to accrual until November 2016. Forty-two potential participants were screened over 24 months, but only 7 were successfully randomly assigned, leading ultimately to the decision to terminate the study. Of the 7 patients recruited, 2 remained in follow-up and 5 had relapsed or died as of February 2020. Despite this, the study made valuable contributions to site infrastructure, and important lessons were learned regarding protocol development and implementation.

## CHALLENGES OF AMC-068

### Protocol Development

AMC-068 was originally conceived as a single-arm, multicenter study intended to further investigate the oCT regimen in a better-characterized patient population that was uniformly treated with concurrent cART. After discussion between investigators and NCI, the study was redesigned as a 2-arm randomized phase II trial to address concerns that the single-arm design would not establish the role of oCT in relation to the regional SOC, CHOP, and that the absence of an SOC arm in the trial was not ethically sound. Additional obstacles during protocol development included limited baseline data to inform study design; lack of consensus among investigators and regulatory bodies regarding appropriateness of the oral regimen and need for randomized comparison with CHOP; and heterogeneity across the SSA sites of standards for diagnosis, staging, and treatment. At the time of initial protocol development, limited published information existed on the epidemiology of AR-NHL in SSA. As highlighted by numerous authors, SSA cancer registries informing data sources, such as GLOBOCAN, have significant limitations,^10^ compounded by deficits in pathology infrastructure. This has contributed to uncertainty of AR-NHL classification, a key issue already encountered in the Mwanda et al^9^ study and also in separate studies in Malawi.^6^

The final challenge was the need to create a protocol that was feasible to implement at multiple sites across SSA. Typically, cancer clinical trial sites in high-income countries have modest differences in local SOC, which can be accommodated within a single clinical trial protocol without undermining the primary objectives. In contrast, the SOC and available resources at the SSA AMC sites varied widely, which changed rapidly, but inconsistently, across all sites. For example, the initial protocol draft assumed no access to hematopoietic colony stimulating factors to manage treatment-related neutropenia. However, after one site indicated that allowing use of granulocyte–colony stimulating factor (G-CSF) was needed to be consistent with their local SOC, the protocol was modified to incorporate 2 separate dose modification schemes for sites with and without G-CSF access. Thereafter, before the protocol was activated, the site that originally requested inclusion of G-CSF declined to participate because persistent concerns of consistency with local SOC, whereas other sites had variable access to G-CSF. By the time the protocol closed, all but 1 site had access to G-CSF, although local protocols for its use continued to vary greatly.

### Pre-Activation

The pre-activation period lasted from final protocol approval in 2012 until the first site activation in 2016, significantly longer than for US-based AMC lymphoma protocols, which historically averaged approximately 100 days. Delays resulted from regulatory approval timelines, establishing and maintaining supply chains, and development of site capacity. Each site had multiple required levels of regulatory approval and an often rapidly changing regulatory environment. In addition, because the study was conducted with US NCI support, each of the local investigators was required to maintain NCI registration, a new and unfamiliar requirement for most SSA investigators.

Although meeting these regulatory requirements took longer than expected, the development and maintenance of a multinational supply chain was even more difficult. Sourcing of study agents was complex and costly. Not all study agents were available in-country. In addition, because of stockouts and unreliable source and quality of locally available drugs, a decision was made to centrally source all study drugs in the United States. Therefore, the AMC needed to maintain a continuous supply of 8 chemotherapeutic agents for each clinical trial site. Furthermore, the cyclophosphamide preparation supplied for the trial required 5% dextrose for intravenous (IV) administration, which was not readily available locally. The chemotherapy supply and procurement processes were complicated by rapid increases in cost of some agents (eg, IV cyclophosphamide), which exceeded the amounts originally budgeted for drugs and required the AMC to solicit donations from pharmaceutical companies. The drug importation process required procurement of documents not normally part of the clinical trial workflow (eg, certificates of analysis) to obtain import permits across multiple countries, while maintaining controlled ambient and cold-chain shipment. Because of delays during this period, donated and purchased pharmaceutical lots expired, and drugs needed replacement. Finally, this period coincided with shortages and escalating costs of specific drugs in the oCT investigational regimen.^11^ We also found supply chains for standard medical supplies (eg, cannulas, infusion lines) were often unreliable, but could be acquired through supply chains within SSA.

### Development of Site Infrastructure

This period was marked by significant investment in site capacity to perform cancer research, building on established clinical and research infrastructure for HIV. Although prospective SSA AMC sites underwent evaluation and audit of readiness for clinical research, the initial site selection process was carried out with an explicit plan for targeted investment to ensure site adherence to international standards. Immunohistochemistry (IHC) expertise and capability, expertise in research pharmacy and nursing, and site-specific policies and procedures for clinical research were developed at all sites through a combination of workshops, facilitated training, ongoing site mentorship, and site audits and evaluations.

Pathology: A significant component of this process was the development of pathology certification, ongoing collaborative pathology reviews, and establishing access to IHC, led by experienced hematopathologists (E.C. and A.C.). Each site was required to have 2 certified pathologists. To qualify, each site pathologist had to show the ability to correctly identify patients with DLBCL based on H&E-stained slide images. To demonstrate proficiency with IHC, each laboratory was required to submit examples of H&E, CD20, and Ki67 slides prepared by the laboratory. Approved pathologists were also required to participate in and present screened study patients on monthly calls led by E.C. and A.C.
Pharmacy: The AMC provided group trainings focusing on pharmacy best practices (eg, familiarity with the US NCI Investigational Agent Drug Accountability Record Form) and engaged an expert South African oncology pharmacist to conduct onsite pharmacy training and review of site-specific pharmacy standard operating procedures (SOPs).
Nursing: Training for chemotherapy nurses was provided by an expert oncology nurse who conducted a centralized training workshop before study activation.
Support for Staging and Response Assessment: To facilitate accurate staging and response assessment, the AMC reimbursed sites for the cost of protocol-mandated computed tomography scans. Training in lymphoma response assessment was provided to investigators by a US-based lymphoma expert as part of study start-up activities.
Site-Specific Implementation Plans and SOPs: Guided development of site-specific SOPs for the research pharmacy, chemotherapy preparation, and administration were used to augment existing clinical pharmacy and nursing services.

### Postactivation

AMC-068 opened to recruitment in Kenya in late 2016. By late 2018, 42 patients had been screened across 4 sites in Kenya, Malawi, Uganda, and Zimbabwe, but only 7 were eligible for randomization. Reasons for ineligibility are presented in Table 2. As shown, of the 35 ineligible patients, the majority were excluded because of non-DLBCL pathology, Ki67 greater than 90%, stage I or II disease, or end-organ dysfunction. Additionally, all sites had some patients who were never screened because of poor performance status or end-organ dysfunction. The mismatch between expected presentation rates of eligible patients with AR-NHL DLBCL versus the actual accrual numbers highlights the impact that unreliable pathology and clinical data can have on study design and implementation in resource-constrained settings. Trials like AMC-068 can begin to bridge those gaps and better inform subsequent trial design. The late presentation arose from poor access of many potential patients to health care facilities and delays from initial presentation of lymphoma symptoms to referral to regional or national centers capable of diagnosing and treating lymphoma. Many of these delays arose from remediable issues such as lack of transport and available funds, and delays in procuring pathology specimens for initial review. On study closure, the AMC study team was continuing to work with sites to establish solutions to these logistic hurdles, including reimbursing sites for the cost of diagnostic biopsies performed as prescreening procedures.

**TABLE 2:**
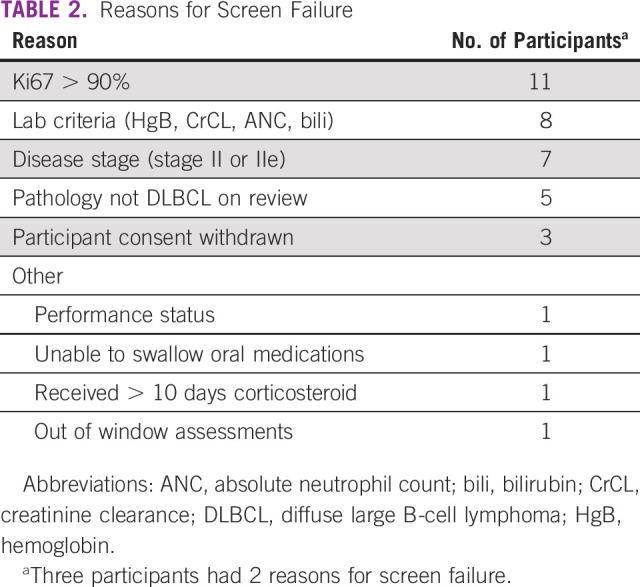
Reasons for Screen Failure

## KEY SOLUTIONS IDENTIFIED

Ultimately, the failure of AMC-068 to recruit eligible patients in a timely manner was due to accrual expectations based on scarce preexisting data and the realities of conducting a complex, multinational, randomized clinical trial in SSA for AR-NHL that met rigorous NCI standards. However, several key insights emerged from this effort.

Multidisciplinary site assessment and support are needed. The original site selection process by the AMC was undertaken by trial operations experts and US-based medical oncologists. Although sites were evaluated by survey and a structured audit tool informed by multiple disciplines (eg, pathology, laboratory services), early engagement at sites by multidisciplinary teams helped identify protocol-specific requirements for site development. As a result, the AMC succeeded in establishing working relationships among oncologists, pathologists, laboratory technicians, pharmacists, study coordinators, and nurses at multiple SSA sites to help assess local requirements, conduct trainings, and identify local solutions when logistical barriers arose. These connections across disciplines have since been leveraged for the successful conduct of other AMC trials in SSA and for the design and implementation of additional protocols currently in development.

Cancer registration and diagnostic facilities in SSA significantly affect the ability to accurately project accrual for clinical trials. Descriptive epidemiology related to cancer burden is highly dependent on pathology and cancer registration infrastructure. GLOBOCAN, a WHO-supported population cancer registry, is cited extensively, but important limitations exist in SSA, where 20 countries lack any registry. Even in countries with existing registries, population coverage ranges from 2.3%-100%, and crude population-level data, even in high-quality registries, often have major limitations with respect to staging or histologic classification,^10^ as is required for clinical trial planning. Contributing to and compounding this uncertainty, pathology across SSA is understaffed and underdeveloped.^12^ In 2013, many countries had < 1 pathologist per million population.^13^ Although AMC-068 sites were intentionally selected partly based on their strong pathology services, the impact of local diagnostic constraints was underestimated initially, and this trial provided a vehicle for working with sites to improve access to and expertise in IHC essential for lymphoma classification.^14,15^ Despite this, delay in referrals to tertiary centers for biopsy often compounded late diagnosis and difficulties in identifying eligible patients. However, AMC-068 served to establish collaboration and certification procedures with site pathologists, improve local IHC capabilities, and facilitate collaborations essential for the eventual creation of a regional biobank for HIV malignancy in SSA,^16^ all of which will support future clinical trials and routine care delivery.^17^

Establishing and maintaining reliable supply chains are critical. AMC-068 faced significant hurdles in procurement of drugs and supplies. During the pre-activation period, it quickly became evident that the time and effort required to oversee the procurement and import/export for these commodities was substantial. To address this, the AMC has developed a local procurement process for drugs and supplies. This approach has clear limitations, including limited availability and stockouts of pharmaceuticals that are well described in SSA,^18,19^ not to mention sometimes unreliable drug quality.^20^ However, when local supply chains for specific, high-quality commodities exist, these can be effectively used and diminish the logistical and administrative burden of conducting cancer clinical trials.

Foreign collaborators must work closely with SSA investigators to understand local SOCs and barriers to trial participation at the individual and health system level. Throughout the process of developing and conducting AMC-068, US and SSA investigators communicated extensively to develop and maintain a shared understanding of local capability and approaches. This was critical because members of the research team had limited experience outside their own health care environment. Creating clear channels for bilateral exchange and dialogue helped bridge these differences to develop a shared strategic vision for priority disease areas and interventions that AMC should pursue in future SSA clinical trials that are both innovative and implementable.

In retrospect, the AMC-068 protocol was an ambitious effort to initiate a multinational, collaborative, randomized clinical trial for AR-NHL at multiple sites in SSA. Because of a mismatch between the protocol-specified population and characteristics of the screened population, this trial was closed early because of poor accrual. However, the effort that went into AMC-068 over many years has yielded strategic insights that will inform the AMC agenda in SSA moving forward and help build a strong operational foundation that will support innovative trials for prevention and treatment of HIV-associated malignancies in SSA for years to come.
